# Shedding Light on the Dynamic Role of the “Target of Rapamycin” Kinase in the Fast-Growing C_4_ Species *Setaria viridis*, a Suitable Model for Biomass Crops

**DOI:** 10.3389/fpls.2021.637508

**Published:** 2021-04-13

**Authors:** Viviane Cristina Heinzen da Silva, Marina C. M. Martins, Maria Juliana Calderan-Rodrigues, Anthony Artins, Carolina Cassano Monte Bello, Saurabh Gupta, Tiago J. P. Sobreira, Diego Mauricio Riaño-Pachón, Valéria Mafra, Camila Caldana

**Affiliations:** ^1^National Center for Research in Energy and Materials (CNPEM), Campinas, Brazil; ^2^Max Planck Institute of Molecular Plant Physiology, Potsdam-Golm, Germany; ^3^Institute of Biochemistry and Biology, University of Potsdam, Potsdam-Golm, Germany; ^4^Bindley Bioscience Center, Purdue University, West Lafayette, IN, United States

**Keywords:** energy sensing, metabolism, biomass, nutrient sensing, C_4_ model, plant growth and development, signaling, target of rapamycin pathway

## Abstract

The Target of Rapamycin (TOR) kinase pathway integrates energy and nutrient availability into metabolism promoting growth in eukaryotes. The overall higher efficiency on nutrient use translated into faster growth rates in C_4_ grass plants led to the investigation of differential transcriptional and metabolic responses to short-term chemical TOR complex (TORC) suppression in the model *Setaria viridis*. In addition to previously described responses to TORC inhibition (i.e., general growth arrest, translational repression, and primary metabolism reprogramming) in *Arabidopsis thaliana* (C_3_), the magnitude of changes was smaller in *S. viridis*, particularly regarding nutrient use efficiency and C allocation and partitioning that promote biosynthetic growth. Besides photosynthetic differences, *S. viridis* and *A. thaliana* present several specificities that classify them into distinct lineages, which also contribute to the observed alterations mediated by TOR. Indeed, cell wall metabolism seems to be distinctly regulated according to each cell wall type, as synthesis of non-pectic polysaccharides were affected in *S. viridis*, whilst assembly and structure in *A. thaliana.* Our results indicate that the metabolic network needed to achieve faster growth seems to be less stringently controlled by TORC in *S. viridis*.

## Introduction

Adaptation and evolution have driven the generation of plants with different metabolism that perform better in particular environments. Plant growth and development are dependent on tightly regulated networks that fulfill the internal demands while responding to external stimuli. One key player integrating nutrient and energy status to control biomass accumulation and metabolism is the serine/threonine Target of Rapamycin (TOR) kinase signaling pathway (Xiong and Sheen, 2012; Dobrenel et al., 2013, 2016b; Saxton and Sabatini, 2017; Shi et al., 2018; Liu and Sabatini, 2020). In most eukaryotes, TOR needs to be assembled into two distinct protein complexes to enable precise substrate recruitment (Schepetilnikov and Ryabova, 2018) to perform particular physiological functions (De Virgilio and Loewith, 2006; Wullschleger et al., 2006; Soulard et al., 2009). In plants, only the TOR complex (TORC) 1 components TOR, RAPTOR, and LST8 have been identified (Menand et al., 2002; Mahfouz et al., 2006; Agredano-Moreno et al., 2007; Maegawa et al., 2015). Despite the conservation of some direct TORC targets from yeasts and animals to photosynthetic organisms (De Virgilio and Loewith, 2006; Wullschleger et al., 2006; Soulard et al., 2009; Dobrenel et al., 2016a), as p70 S6 Kinase (S6K/Sch9) involved in protein translation, other targets are either missing or new regulatory steps were aggregated according to the complexity level of organisms for fine-tuning TOR activity (Henriques et al., 2014; Xiong and Sheen, 2015; Shi et al., 2018). Thus, the sessile life form of plants, besides their photoautotrophic nature, provides a special niche for the discovery of unknown mechanisms under the coordination of this pathway.

TOR is a two-fold controller regulating the production of several building blocks (Saxton and Sabatini, 2017) at the same time that mediates cell proliferation (Henriques et al., 2010; Xiong and Sheen, 2012; Barrada et al., 2019) being crucial for keeping the cellular homeostasis to sustain growth. In plants, growth is defined by a permanent increase in size, involving changes in dry weight (biomass), cell expansion (extension), and division (proliferation). These processes are interdependent, as metabolic activity drives biomass accumulation through the acquisition of nutrients and photosynthetic fixation of carbon dioxide (CO_2_). TOR senses carbon (C) availability to modulate developmental programs, ensuring the balance between the source supply and sink demand at different stages of plant growth (e.g., Dobrenel et al., 2013; Xiong et al., 2013; Pfeiffer et al., 2016; Zhang et al., 2016; Brunkard et al., 2020). TORC seems to perform conserved functions in plants, particularly affecting the efficiency of C assimilation, partitioning, and use for growth. For example, TORC adjusts various primary metabolic routes and coordinates C partitioning between growth and storage molecules in algae and the eudicot Arabidopsis *thaliana* (C_3_) (Moreau et al., 2012; Ren et al., 2012; Lee and Fiehn, 2013; Caldana et al., 2013; Jüppner et al., 2018; Salem et al., 2018; Pancha et al., 2019). However, the upstream and downstream players might have evolved differently according to the C demands of the tissue type.

Photosynthesis represents the only source of C for the generation of organic molecules used according to the rates of cell division/expansion and developmental transitions (Smith and Stitt, 2007; Siqueira et al., 2018; Wingler, 2018). Different photosynthetic mechanisms exist in plants, in which enzymes from distinct metabolic pathways can fix C into C_3_ or C_4_ acids. In most plant species, referred to as C_3_, ribulose-1,5-bisphosphate carboxylase/oxygenase (RubisCO) catalyses C fixation into 3-phosphoglycerate (3PGA) via the Calvin Benson cycle. This reaction is limited by the activity of RubisCO as a result of the slow catalytic turnover and its oxygenase reaction that diverts the C flow through photorespiration (Stitt et al., 2010; Raines, 2011). C_4_ plants have a biochemical CO_2_ concentrating mechanism in which C is initially fixed by phosphoenolpyruvate carboxylase into oxaloacetate (OAA) in the mesophyll cells. OAA is then transported and decarboxylated into 3PGA in bundle sheath cells increasing CO_2_ concentration for RubisCO (Furbank, 2017). This complex set of biochemical and anatomical specializations brought about improved C gain, nitrogen (N), and water use efficiencies boosting the growth rates (Atkinson et al., 2016; Furbank, 2017; Sage et al., 2018; Leakey et al., 2019). Furthermore, due to their less dense tissues, C_4_ species optimize their C allocation producing more leaves and roots than C_3_ (Atkinson et al., 2016).

The precise regulation of the partitioning of photosynthetic products into different functional C pools (C allocation) is one of the main determinants for controlling biosynthetic growth, and therefore, biomass. Photoassimilates produced in the source leaves during photosynthesis are translocated through the phloem and imported into non-photosynthetic C-consuming sinks (e.g., roots) for generating energy to fuel metabolism (consumption) or growth (storage) (Chang and Zhu, 2017). When sucrose production in source leaves exceeds sink demand, sucrose accumulation leads to a feedback inhibitory effect on photosynthesis switching photoassimilate partitioning into C reserves such as starch (Heyneke and Fernie, 2018). This mechanism coordinates photosynthesis and sink demand to control growth in C_3_ plants. Recent work suggested that this feedback regulation might operate differently in C_4_ plants (Henry et al., 2020). Despite increased sugar levels in plants grown under high light conditions, photosynthesis and transcriptome of the C_4_ grass *Setaria viridis* source leaves responded more dramatically to low light (Henry et al., 2020). The authors found that TOR was affected neither by light intensity nor by sugar content at the transcriptional level in this organ. TOR transcript levels are quite stable to environmental changes even in the C_3_ *A. thaliana*, the plant species with most research on TOR.

In this work, we used *S. viridis*, the photosynthetic model for high biomass panicoid crops (Martin et al., 2016; Mamidi et al., 2020), to explore the consequences of TOR repression and included *A. thaliana* only as a standard for comparison. These species are not only contrasting in terms of photosynthetic metabolic pathways, but they also belong to different lineages presenting morphological specificities in their root (e.g., general root and hair pattern) and leaf architecture (e.g., vasculature pattern) (Hochholdinger and Zimmermann, 2008; Nelissen et al., 2016; Conklin et al., 2019). *S. viridis* is closely related to agronomically important C_4_ crops with the NADP-malic enzyme (NADP-ME) subtype, such as sorghum, sugarcane, and maize and it has been suggested as a convenient genetic model to study C_4_ plants due to its short life cycle, sequenced genome, and transformability (Brutnell et al., 2010; Petti et al., 2013; Huang et al., 2016). Chemical inhibition of TOR by AZD8055 followed fine-kinetic modifications on transcripts and primary metabolites during the day, the period in which differential C assimilation occurs. In addition, root growth and biomass accumulation were evaluated in longer periods of drug exposure. Our results confirm the role of TORC in controlling plant growth also in the faster-growing grass *S. viridis*, however, the observed responses were overall mildly impacted by this signaling pathway.

## Materials and Methods

### Protein Sequences, Alignment, and Domain Prediction

Protein sequences of TOR or FKBP12 were retrieved from National Center for Biotechnology Information (NCBI)^1^ and Phytozome12 (Phytozome, RRID:SCR_006507) databases (Supplementary Tables 1–3). *A. thaliana* sequences were defined as a query for all analyses. Alignments were generated by Clustal Omega 2.1^2^ (Clustal Omega, RRID:SCR_001591), whereas domain- and motif-like patterns were predicted with Motif^3^ and InterProScan^4^ (InterProScan, RRID:SCR_005829) softwares, respectively.

### Yeast Complementation Assays

The FKBP12 sequences from *S. viridis* and *A. thaliana* (Supplementary Table 2) were amplified by PCR with primers listed in Supplementary Table 3 and checked by sequencing. The resulting PCR products were cloned by homologous-end recombination (Oldenburg et al., 1997) into the yeast PWS28 vector (Mokry et al., 2009). *Saccharomyces cerevisiae* wild-type BY4741 or the mutant strain lacking FKBP12 (*fpr1*, Euroscarf) were transformed (Elble, 1992) with the empty PWS28 vector or with the different versions of PWS28-FKPB12 constructs and plated onto synthetic dextrose solid media lacking uracil (SD-Ura) (Sherman, 2002). Complementation assays were performed by serial dilution (1:10) of yeast cultures with an OD_600_ of 0.6 spotted (2.5 μl) onto SD-Ura media supplemented with the final concentrations of rapamycin. The plates were incubated for 2 days at 30°C. For determination of doubling times, yeasts were grown in SD-Ura liquid media supplemented with 1 μM rapamycin in 96-well plates at 30°C and OD_600_ was measured every 15 min. Growth curves were plotted as log_10_OD_600_ in the function of time, and the slope values were obtained from the linear regression of the log phase for each strain. Peak doubling times for the cultures were determined by the equation Td = log_2_/a, where Td is the time of duplication, and “a” is the slope value. ANOVA-test (*P* < 0.05) was used to assess statistical significance in the growth of the different yeast strains.

### Plant Material and Growth Conditions

**FIGURE 1:**
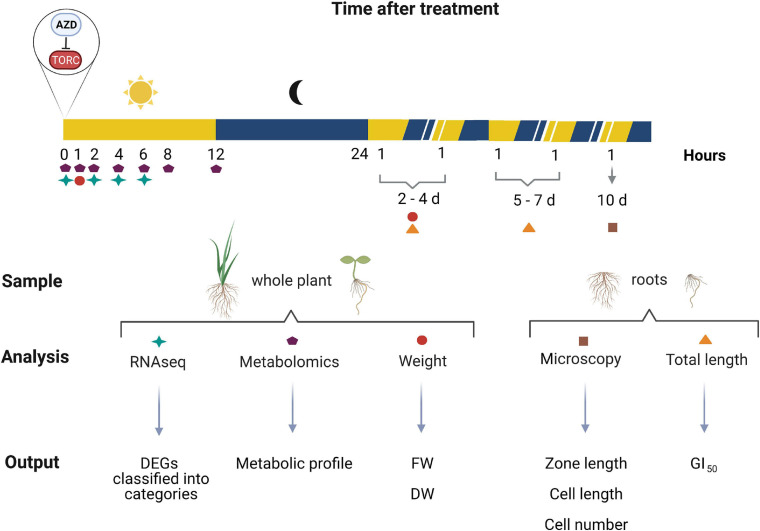
Overview of the experiments performed in this study. Created with BioRender.com.

Seeds from *S. viridis* [L.] Beauv (accession A10.1) and *A. thaliana* [L.] Heynh. (accession Columbia-0) were surface sterilized and, unless otherwise stated, cultivated on a hydroponic system using tip boxes (Monte-Bello et al., 2018) in half-strength MS medium with vitamins (Murashige and Skoog, 1962) in 12 h photoperiod. Seeds were germinated in controlled growth chambers (fitotron^®^ models HGC 1514 and SGC 120, Weiss Technik) under 75% of humidity, 300 μmol m^–2^ s^–1^ of irradiance and temperature of 28°C (day)/25°C (night) for *S. viridis* or 150 μmol m^–2^ s^–1^ of irradiance and temperature of 21°C (day)/19°C (night) for *A. thaliana*. After reaching stages 11 (*S. viridis*) and 1.04 (*A. thaliana*) from the BBCH scale (Boyes et al., 2001; Rahbari et al., 2012; Hodge and Doust, 2017), the hydroponic tanks were replaced with a fresh medium containing 0.05% DMSO (control), 10 (*S. viridis*) or 2 μM (*A. thaliana*) AZD8055 (LC Laboratories) 30 min before the beginning of the light period. This DMSO concentration is within the ideal range of most of the experiments evaluating TORC function in plants (Montané and Menand, 2013, 2019; Ouibrahim et al., 2015; Prioretti et al., 2017; Van Leene et al., 2019). An overview of all experiments is provided in Figure 1. For molecular and metabolic profiling analysis, whole seedlings were harvested at each time point/condition, immediately frozen in liquid nitrogen, and stored at −80°C until use.

Total fresh weight (FW) and dry weight (DW) of seedlings were determined on an analytical balance (accuracy 0.0001) along 4 days of treatment with DMSO 0.05% (control), 10 or 2 μM AZD8055 (*S. viridis* and *A. thaliana*, respectively). To avoid errors in estimating the weights of single plantlets, pools of 5 and 15 seedlings of *S. viridis* and *A. thaliana*, respectively, were weighed as a single replicate in a total n of 5 or 4 pools. The values of each pool were divided by the number of plants in the respective pool and the averages ± standard error were calculated. This was done to reduce the bias toward the number of seedlings for each species. Significant differences along time within the same treatment were evaluated using ANOVA and are indicated by letters (*P* < 0.05). Significant differences between treatments (DMSO or AZD8055) were analyzed by Student’s *t*-test and are indicated by asterisks (*P* < 0.05).

### Root Phenotyping

For assessing the effect of TOR inhibitors on *S. viridis* root growth, surface-sterilized seeds were germinated on vertical plates containing solid half-strength MS medium with vitamins in the same conditions described in the previous section. After reaching the developmental stages described in plant material and growth conditions, seedlings were transferred to plates containing the same media supplemented with DMSO 0.05% (control), AZD8055 or rapamycin (10 μM). Root length (*n* = 40 *S. viridis* roots) was measured for 7 days of cultivation and the average was calculated. Significant differences between the treatments were analyzed by ANOVA and are indicated by letters (*P* < 0.05). The same conditions were used to evaluate the effect of different AZD8055 concentrations, ranging from 0 to 10 μM, on *S. viridis* and *A. thaliana.* Root growth (*n* = 15 seedlings for *S. viridis* or *A. thaliana*) was evaluated for 7 days by calculating the root inhibitory dose according to (Montané and Menand, 2013).

Root tips of seedlings (*n* = 4 and 5 seedlings for *S. viridis* and *A. thaliana*, respectively) treated for 10 days with DMSO 0.05% (control), 10 or 2 μM AZD8055 (*S. viridis* and *A. thaliana*, respectively) were fixed in 12.5% acetic acid in ethanol for 1 h and sequentially washed with 100% ethanol, 50% ethanol, and demineralized water. Roots were maintained in 1 mM KOH at 4°C, mounted in demineralized water, and imaged using a conventional light microscope coupled with a differential interference contrast system (BX51 and Olympus) at magnification x40. Measurements in epidermal cells of root surface were performed with ImageJ software taking into account the distance of cells from the quiescent center (QC). Only a single line of cells was measured along the root toward the maturation zone, and the position of each cell was defined by its endpoint. Meristematic and elongation zone sizes were defined by the distance between QC and the first elongated cell, and the distance between the first and last elongated cells, respectively. To estimate the number of cells in the meristematic zone, the length of the zone was divided by the average meristematic cell length. Significant differences between the treatments were assessed by Student’s *t*-test and are indicated by asterisks (*P* < 0.05).

### Metabolite Profiling Analysis

Primary metabolites were extracted from 20 mg of plant material (*n* = 5 replicates composed by pools of 15 or 35 *S. viridis* or *A. thaliana* seedlings, respectively) using methyl-tert-butyl-ether extraction (MTBE) buffer (Giavalisco et al., 2011). Polar fractions were concentrated, derivatized with N-methyl-N-trimethylsilyltrifluoroacetamide, and analyzed by gas chromatography (GC) (7890N and Agilent) coupled to time-of-flight (TOF) mass spectrometry (MS) (Pegasus HT and Leco) (Lisec et al., 2006). Peak detection, retention time alignment based on FAMEs, and mass spectral comparison with reference libraries were performed using TargetSearch (Cuadros-Inostroza et al., 2009). Metabolite identification was also manually supervised. Metabolites were quantified based on the peak intensity for a selected mass and subsequently normalization to the sample FW and total ion count, and log_2_ transformed. Data normalization and statistical analysis were performed in R v3.2.2^5^. Pairwise comparisons of metabolites between control and AZD8055-treated samples at each time point were calculated using Student’s *t-*test (*P* < 0.05). Principal component analysis (PCA) was carried out using pcaMethods R package (Stacklies et al., 2007) and heatmaps generated in Excel using a macro (Gibon et al., 2006) (Supplementary Datasets 1–3).

For starch quantification, the insoluble material remaining after the MTBE extraction was solubilized in 0.1 M NaOH by heating to 95°C, neutralized, digested enzymatically overnight and the released glucose was then used to determine starch content of the samples spectrophotometrically by coupling it to the reduction of NADP^+^ to NADPH (Hendriks et al., 2003). The same statistical analysis performed for metabolomics was used for starch content.

### RNA Preparation and Transcript Expression Profiling

One μg of total RNA extracted (*n* = 3 replicates composed by pools of 15 or 35 *S. viridis* or *A. thaliana* seedlings, respectively) using SV Total RNA Isolation System (Promega) was used to generate libraries according to TruSeq Stranded mRNA HT Sample Prep Kit (Illumina). The size and quality of libraries were confirmed with the 12,000 DNA assay kit (Agilent) and quantification performed with the KAPA Library Quantification Kit Illumina^®^ Platforms (Kappa Biosystems). Libraries were pooled in equimolar ratios and submitted to paired-end sequencing on a Hiseq 2500 (Illumina) using TruSeq High Output SBS Kit v3 – HS (Illumina) at the Brazilian Bioethanol Science and Technology Laboratory (CTBE/CNPEM). Raw sequencing reads were trimmed using Trimmomatic v0.38 (Bolger et al., 2014) (Trimmomatic, RRID:SCR_011848), followed by further filtering to remove rRNA contamination using SortMeRNA v2.1 (Kopylova et al., 2012) (SortMeRNA, RRID:SCR_014402). Quantification was performed with kallisto v0.44.0 (Bray et al., 2016) (kallisto, RRID:SCR_016582) against cDNA sequences (*A. thaliana*: Araport11 – Cheng et al., 2017; *S. viridis*: v2.1 from Phytozome12). An average of 51.9 and 13.3 million reads were obtained for *S. viridis* and *A. thaliana*, respectively, whereby about 5% were discarded after quality trimming and rRNA removal, and more than 95% mapped to the annotated genome for each species (Supplementary Dataset 4). Differential expression analysis was carried out using EdgeR package (Robinson et al., 2010) (edgeR, RRID:SCR_012802) as well as cross-sample normalization to obtain trimmed mean M-value (TMM) normalized counts. Thresholds of | log_2_ fold change| ≥1 and FDR < 0.05 were used to identify differentially expressed genes (DEGs) (Supplementary Datasets 5–7). Mercator4 v1.0^6^ (Lohse et al., 2014) was employed to annotate the coding sequences of *S. viridis* with MapMan bins (Thimm et al., 2004) (MapMan, RRID:SCR_003543) (Supplementary Dataset 8). RNA-seq can be found under accession number BioProject ID PRJNA494848 in the NCBI SRA database^7^. The Arabidopsis DEGs after AZD8055 treatment were compared with three previous published datasets (Ren et al., 2012; Xiong et al., 2013; Dong et al., 2015) using the function merge in R (Supplementary Dataset 9).

## Results

### AZD8055 Inhibits Root Growth of *S. viridis* Seedlings in a Dose-Dependent Manner at Higher Concentrations Than of *A. thaliana*

Sequences of the TOR protein were first compared among selected photosynthetic organisms, from the green algae *Chlamydomonas reinhardtii* to monocots and eudicots. As previously found for *Setaria italica* (Sapre et al., 2018), *S. viridis* TOR protein sequence is also well-conserved to plants (Supplementary Figure 1), yeast, and humans. Monocots present a more relaxed leucine zipper (leu zip) (position 1028–1050 in Arabidopsis) as the third leucine is replaced by valine (data not shown). This leu zip is responsible for DNA binding activating the 45S rRNA promoter and expression of rRNA (Ren et al., 2011). The impact of this looser structure needs further investigation.

The FRB domain of TOR forms a ternary complex with the immunophilin protein FKBP12 and the rapamycin, repressing only the effectors of TORC1 (Chiu et al., 1994; Sabatini et al., 1994). In plants, the lack of critical amino acids residues in FKBP12 hampers the formation of a stable ternary complex with rapamycin and TOR (Sormani et al., 2007; Leiber et al., 2010; Ren et al., 2012; Xiong and Sheen, 2012). A slightly higher affinity of the complex to rapamycin has been previously reported for maize (Agredano-Moreno et al., 2007) as a consequence of an amino acid substitution, which is also present in *S. viridis* FKBP12 sequence (S-55, Supplementary Figure 2A). To investigate the role of this residue in the ternary complex stabilization, we performed an *in vivo* experiment using yeast complementation assay. SvFKBP12 protein could weakly complement the *fpr1*Δ strain grown under 1 μM rapamycin when compared with the same strain transformed with the empty vector (Supplementary Figure 2B) but presented a much lesser effect than the strains transformed with the ScFKBP12. When the growth curves of these strains were performed, rapamycin-treated *fpr1*Δ strains overexpressing SvFKBP12 presented an increase of 1.3 h in their doubling time during the exponential phase in relation to the strain harboring the empty vector, indicating a slight decrease in cell proliferation (Supplementary Figures 2C,D). Similarly, roots of *S. viridis* seedlings treated with 10 μM rapamycin also presented a tendency to reduce growth after 3 day of treatment (Supplementary Figure 2E) but this decrease in root length was not always reproducible in independent experiments. It remains to be investigated whether the substitution of the residue S-55 might enable a slightly better affinity to the drug than the corresponding A-58 found in *A. thaliana* (Menand et al., 2002; Mahfouz et al., 2006; Sormani et al., 2007; Ren et al., 2012; Deng et al., 2016). However, S-55 itself does not seem to confer stability to this ternary complex, precluding the use of rapamycin to investigate the TOR pathway in *S. viridis*.

**FIGURE 2:**
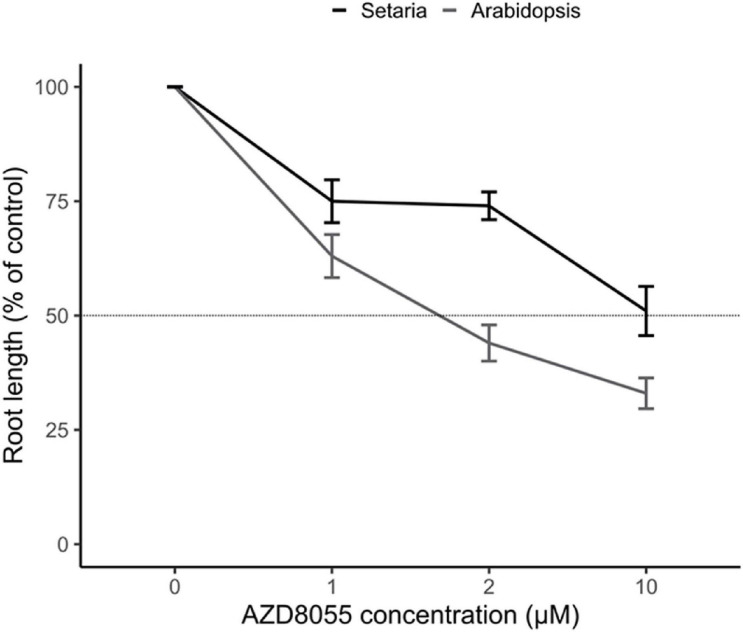
TOR effect on root growth inhibition in *S. viridis* and *A. thaliana*. Seedlings of *S. viridis* and *A. thaliana* were grown under 12 h photoperiod until specific and compatible developmental stages before the transference to plates containing different concentrations of AZD8055 (1–10 μM) or DMSO 0.05% (control). The effect of AZD8055 on root growth (*n* = 15 roots for *S. viridis* or *A. thaliana*) was expressed relative to DMSO 0.05% to identify the AZD8055 inhibitory concentration (GI_50_) according to Montané and Menand (2013).

AZD8055 is a widely used active-site TOR inhibitor operating directly at the ATP-binding pocket competing for substrate phosphorylation for both TORC1 and TORC2 in humans (Chresta et al., 2010) and has been extensively used for studying the role of this complex in plants (Montané and Menand, 2019 and references therein). As plant growth inhibition by ATP-competitive TOR-inhibitors (asTORis) is dose-dependent (Montané and Menand, 2013), we next examined the *in vivo* sensitivity of *S. viridis* and *A. thaliana* to AZD8055. When concentrations of this drug ranging from 1 to 10 μM were tested, root growth was impaired in both species (Supplementary Figures 3A,B). However, the dose-response curves revealed that 50% primary root growth-inhibitory dose (GI_50_) was reached after 2 days of treatment with 2 μM AZD8055 for *A. thaliana*, whereas *S. viridis* needed a concentration five-fold higher to exert the same effect (Figure 2). *S. viridis* seedlings have three to five-fold higher FW than *A. thaliana* depending on the evaluated time point (Supplementary Figures 3E,F), which reflected the use of 10 μM AZD8055 to compare the effects of TOR repression. This resembles the AZD8055 concentration that inhibits root growth when comparing *A. thaliana* to the C_4_ *Panicum miliaceum* (Montané and Menand, 2013), pointing out the degree of response concerning the plant biomass accumulation.

**FIGURE 3:**
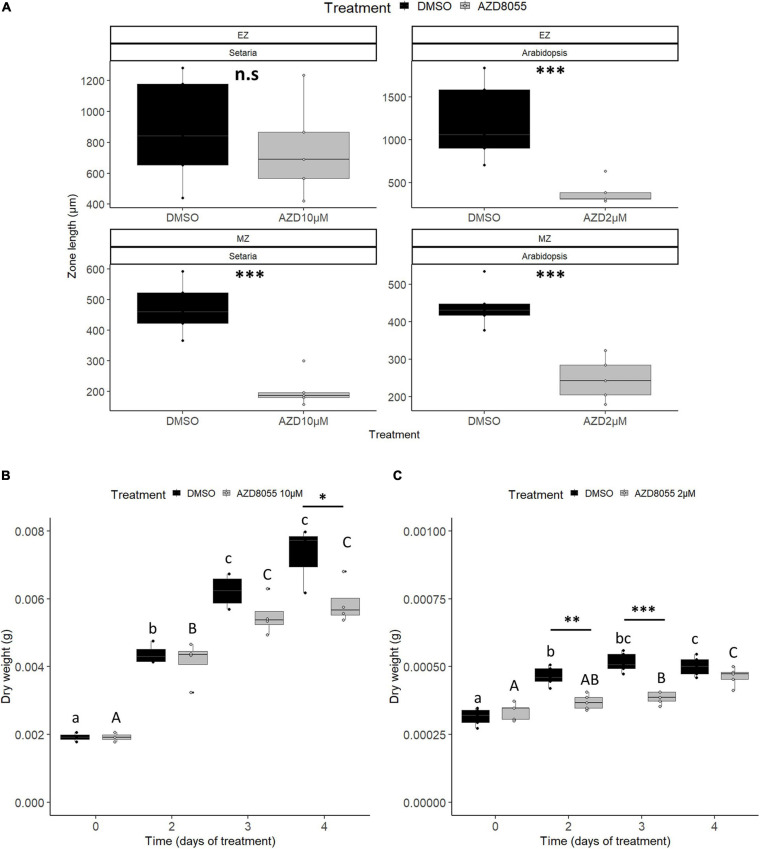
AZD8055 severely impacts the growth of *S. viridis* and *A. thaliana* seedlings. Effect of AZD8055 on root and overall growth of *S. viridis* and *A. thaliana*. For all measurements, seedlings were grown hydroponically under 12 h photoperiod until specific and compatible developmental stages before application of DMSO 0.05% (control), 10 or 2 μM AZD8055 (*S. viridis* and *A. thaliana*, respectively). **(A)** Length of root EZ and MZ, expressed in cm. DW of *S. viridis.* **(B)** and *A. thaliana.* **(C)** Black and gray colors represent DMSO and AZD8055 treatments, respectively. Significant differences along time within the same treatment, using ANOVA, are indicated by letters (*P* < 0.05), lower case for DMSO and capital letters for AZD8055-treated plants, and significant differences between treatments are indicated by asterisks (Student’s *t*-test): ^∗^*P* < 0.05, ^∗∗^*P* < 0.01, and ^∗∗∗^*P* < 0.001.

Longitudinally, primary roots can be divided into three zones: (*i*) meristematic (MZ), where rapidly proliferation occurs; (*ii*) elongation (EZ), where cell division ceases and elongation takes place; and (*iii*) differentiation/maturation (MatZ), where cells achieve their final shape and size and can differentiate into secondary organs (reviewed by Barrada et al., 2015). Ten days of AZD8055 treatment brought about a significant decrease in MZ length of root epidermis in *A. thaliana* and *S. viridis* (Figure 3A), also observed in *raptor1b* (Salem et al., 2018). As roots were swollen after the treatment, cell length and cell number were monitored instead of meristematic cell area. Increased meristematic cell length followed by reduced cell number was detected after TORC repression (Supplementary Figures 3C,G), further indicating impaired cell proliferation in both species. Interestingly, EZ length was reduced (ca. 70%) in *A. thaliana* roots, but no significant alteration was found in *S. viridis* (Figure 3A). As MatZ cell length was significantly decreased in both species after treatment (Supplementary Figure 3D), the smaller impact on EZ length in this grass might indicate a narrower window of the AZD8055 effect and faster recovery, which might be linked to the species growth rate. The root architecture in monocots and eudicots are diverse, and the smaller impact of AZD8055 on *S. viridis* cell length might indicate different strategies as its fibrous roots are more prone to ramification instead of investing in one main root (reviewed by Hochholdinger and Zimmermann, 2008).

Concerning growth in terms of biomass acquisition, *S. viridis* seedlings displayed a significant daily increment of FW and DW in the control, whereas in *A. thaliana* these differences were observed only after 3 and 2 days of transference to the new media containing DMSO, respectively (Figures 3B,C; Supplementary Figures 3E,F), compatible with the faster-growing ability typical of C_4_ grass. When seedlings were subjected to AZD8055 treatment, *A. thaliana* FW declined in relation to the control after 4 days (^∗∗∗^*P* < 0.001) and *S. viridis* has a faster but less significant effect at 3 days (^∗^*P* < 0.05) (Supplementary Figures 3E,F). On the other hand, treatment with AZD8055 significantly decreased DW earlier in *A. thaliana* compared with *S. viridis* (Figures 3B,C). Interestingly, although the drug concentrations applied were able to influence root growth similarly after 2 days (Figure 2), DW data suggest that 2 μM AZD8055 exerted a more severe biosynthetic growth reduction in *A. thaliana* than 10 μM AZD8055 in *S. viridis*.

### Primary Metabolism Is Markedly Less Affected by TORC Repression in *S. viridis*

The milder changes in *S. viridis* DW, which directly reflects C incorporation into biomass, prompted us to investigate to which extent AZD8055 treatment affected its metabolism. We profiled primary metabolites using GC-TOF-MS in seedlings of both species exposed to short-term AZD8055 treatment. Seedlings were harvested at 0 (i.e., 30 min before the light was switched on), 1, 2, 4, 8, and 12 h of AZD8055 or DMSO treatment (Figure 1). This period covers part of the diel cycle when photosynthetic C assimilation occurs and most of the cell biosynthetic blocks are synthetized. A total of 61 (*S. viridis*) and 49 (*A. thaliana*) compounds with known chemical structures were determined, 42 of which were common to both species (Supplementary Dataset 1).

We performed a principal component (PC) analysis to compare AZD8055 treatment across time in both plant species (Supplementary Figure 4 and Supplementary Dataset 2). PC1 (55 and 46% of the total variance in *S. viridis* and *A. thaliana*, respectively) separated samples along the first half of the diel cycle in both species. The responses at time point 0 h represent the metabolic status at the end of the night/darkness period. Hexoses and quinate contributed to the separation toward the time of exposition to light in both species. It is reasonable that due to its higher growth rate, the primary metabolism of *S. viridis* would respond more intensely and promptly to light. Indeed, the separation was greater between the samples at 0 h and the remaining time points in this grass. Most of the amino acids have increased and reduced levels at dawn in *S. viridis* and *A. thaliana*, respectively. In *S. viridis*, uracil, serine (Ser), lysine, and isoleucine led to the discrimination of the samples at this time point. At the end of the night, metabolic responses are well-characterized in *A. thaliana*, when plants have almost exhausted their C reserve storage in the form of starch to provide sugars and supply growth (Smith and Stitt, 2007). In agreement, our data showed that maltose, the major product of starch breakdown, together with orthophosphate, arginine (Arg), and ornithine (Orn) contributed to the sample separation at 0 h in *A. thaliana*.

In PC2 (15% and 20% of the total variance in *S. viridis* and *A. thaliana*, respectively), the samples were grouped according to the treatment. Although the compounds driving this separation were distinct, metabolites involved in C storage and transport contributed to the discrimination of the control samples in both species. In *S. viridis*, fructose, sucrose, fumarate, and ketoglutarate were important for the segregation of the control samples over time. Fructose and sucrose can act as the main C source to drive energy production, metabolism, and the synthesis of building blocks (e.g., proteins and DNA) in cell-proliferating tissues in *S. viridis* (Martin et al., 2016). In addition, sucrose and C_4_ acids, like fumarate, can play a role as C storage compounds in this C_4_ model grass. On the other hand, starch content rises due to AZD8055 treatment in *A. thaliana* and is not significantly altered in *S. viridis* (Supplementary Figure 5). In *A. thaliana*, glycine, Arg, Orn, galactinol, and raffinose were the main drivers for separating the control samples over time. As previously reported (Caldana et al., 2013; Salem et al., 2018), branched-chain amino acids, tyrosine, and citrate separated the TOR inhibited samples in *A. thaliana*, while isocitrate, glutamine, galactinol, and dehydroascorbate (DHA) were important for the discrimination of AZD8055-treated samples in *S. viridis*.

**FIGURE 4:**
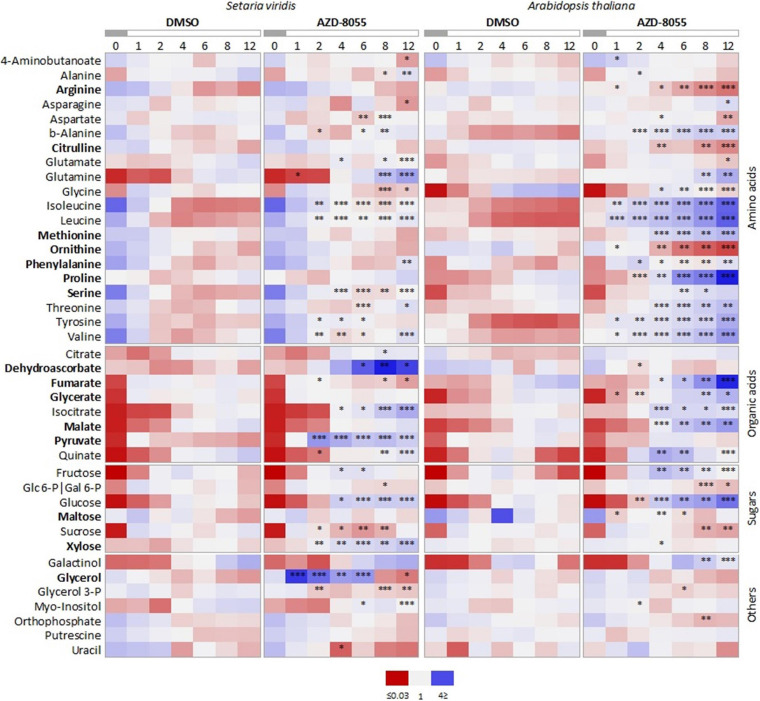
Comparative heatmap of metabolic changes in *S. viridis* and *A. thaliana* seedlings under TORC inhibition. Seedlings were grown hydroponically under 12 h photoperiod until specific and compatible developmental stages before application of DMSO 0.05% (control), 10 or 2 μM AZD8055 (*S. viridis* and *A. thaliana*, respectively). Metabolite profiling was carried out using GC-TOF-MS. Data represents the average of biological replicates (*n* = 5), median scaled, and normalized log_2_-transformed values. Significant differences between metabolites from control and TOR-inhibited seedlings are indicated by asterisks (Student’s *t-*test): ^∗^*P* < 0.05, ^∗∗^*P* < 0.01, and ^∗∗∗^*P* < 0.001, and also available on Supplementary Dataset 3. Metabolites in bold indicate differential behavior in *S. viridis* and *A. thaliana*.

We performed a heatmap using the relative levels of metabolites commonly identified for both species (Figure 4 and Supplementary Dataset 3) to closely investigate their patterns along with the time series. Most of the compounds that respond to AZD8055 treatment in *A. thaliana* tend also to have their levels altered in *S. viridis*, but with a lower magnitude. Since these changes were extensively reported previously (Moreau et al., 2012; Caldana et al., 2013; Salem et al., 2017), we focused only on the metabolites with specific behavior upon TOR inhibition in *S. viridis*. These were DHA, fumarate, and xylose. The increased levels of DHA might indicate alterations in the cellular redox state important to relieve stress. Reduction in xylose, the main component of the hemicellulose xylan abundant in grasses, and in fumarate might suggest less incorporation into cell wall and limited use of this alternative C sink for respiration, respectively. Therefore, these results could strengthen the negative impact of TOR repression on *S. viridis* growth and development (Figures 2, 3B).

Taken together, TOR inhibition triggers common changes in *S. viridis* metabolism as the ones previously reported for other photosynthetic organisms (Moreau et al., 2012; Ren et al., 2012; Caldana et al., 2013; Salem et al., 2017; Jüppner et al., 2018; Mubeen et al., 2018, 2019). These include alterations in C and N metabolism that impact the production of building blocks, growth, and biomass incorporation. Following the same tendency observed in the DW, metabolic changes are affected to a lesser extent in this grass. Thus, metabolic regulation under TORC-dependent control seems to be less stringent than in other organisms, at least under our experimental conditions.

### Short-Term TORC Inhibition Has a Far-Reaching Effect on the Transcriptome, Less Pronounced in *S. viridis*

**FIGURE 5:**
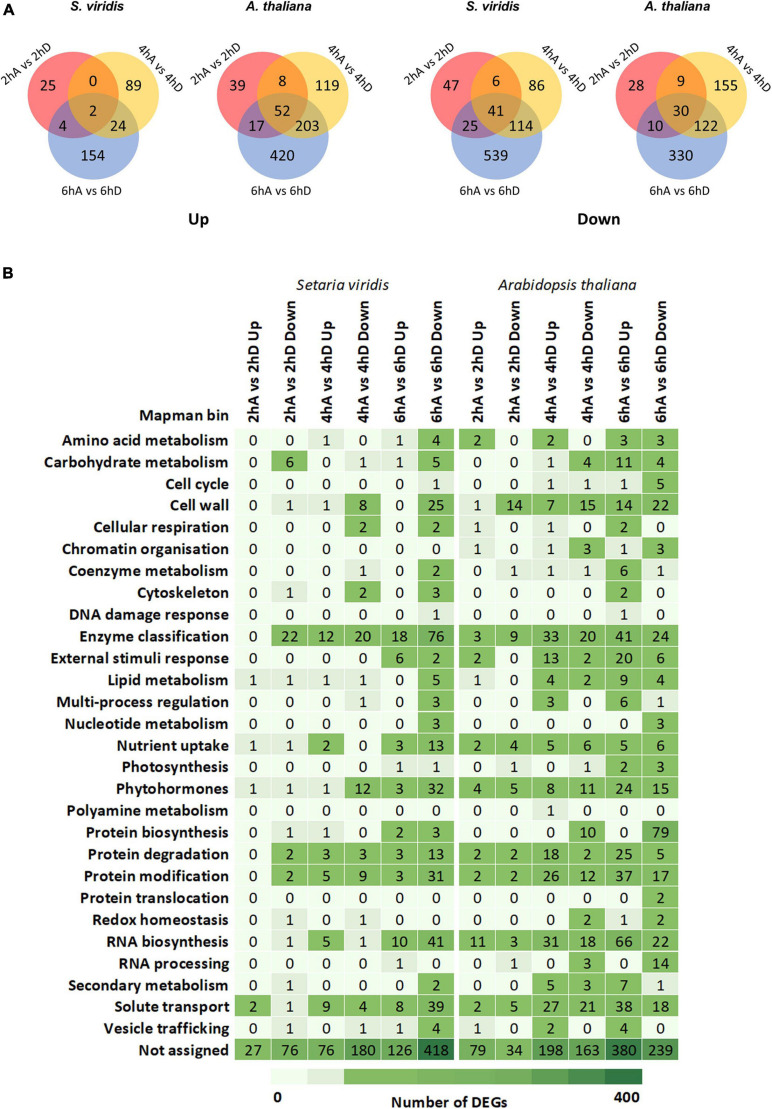
Comparative transcriptional changes in *S. viridis* and *A. thaliana* under TOR inhibition. **(A)** Venn diagrams of DEGs among distinct time points under TORC inhibition in *S. viridis* and *A. thaliana*, showing down- and up-regulated genes. **(B)** Classification of DEGs into Mapman categories in *S. viridis* and *A. thaliana* seedlings under TORC inhibition. The list of DEGs from control and TOR-inhibited seedlings is available on Supplementary Dataset 8.

To gain further insight into the biological processes involved in the short-term response of TORC inhibition, we performed RNAseq analysis in seedlings harvested at 0 (i.e., 30 min before the light was switched on), 2, 4, and 6 h of AZD8055 or DMSO treatment (Figure 1). A total of 25% of the mapped genes were DEGs (log_2_FC ≥ 1 and FDR < 0.05) in *S. viridis* and *A. thaliana*, when summing up the differentially expressed transcripts at all time-points (with and without treatment) relative to time point 0 (Supplementary Dataset 7). These large changes were accompanied by alterations in primary metabolites (Figure 4) and were expected as light, diel, and circadian cycles have a major impact on transcriptional and metabolic networks (Harmer et al., 2000; Bläsing et al., 2005; Casal and Yanovsky, 2005; Gibon et al., 2006; Espinoza et al., 2010; Caldana et al., 2011; Ruckle et al., 2012). When only the effect of treatment was considered, 1,542 and 1,156 DEGs were detected in the C_3_ and C_4_ models, respectively, regardless of the time point and, as expected, with a larger number of transcripts changing over time (Figure 5A). Thirty-seven percent of Arabidopsis DEGs (567 transcripts) identified in this work displayed a similar expression pattern with at least one of the previously reported studies using different strategies to investigate the consequences of TOR repression in this species (Ren et al., 2012; Xiong et al., 2013; Dong et al., 2015) (Supplementary Dataset 9). This overlap includes genes belonging to categories well-known to be controlled by the TOR pathway, such as translation, ribosome biogenesis, cell wall, and cell cycle. Not surprisingly, the largest overlap in DEGs, 241 up- and 265 down-regulated genes, was found comparing our results with those of Dong et al. (2015), which also assessed the response of AZD8055 treatment in seedlings, although at later time points (24 h). Overlaps to some extent were also shown in contrasts with transgenic lines, estradiol-inducible *tor* RNAi seedlings at the heterotrophic to photoautotrophic transition (72 up- and 164 down-regulated genes, Xiong et al., 2013) and rapamycin-sensitive (84 up- and 33 down-regulated genes, Ren et al., 2012). Despite the differences in the experimental setup including duration of the TOR repression, growth conditions (e.g., photoperiod and exogenous sucrose supply), and transcriptomics platforms, our analysis further proposes the presence of certain conserved modules of TOR regulation in *A. thaliana*.

Relevant biological processes affected by TORC repression were explored with MapMan (Thimm et al., 2004). Individual DEGs for each pairwise comparison (e.g., 2 h DMSO versus AZD8055) were analyzed independently for each species. Out of 35 bins, 28 were found at least in one pairwise comparison per species, but a large proportion of DEGs was not assigned to any particular process (Figure 5B). This classification did not limit the broad discussion of interconnected biological processes. In general, categories such as cell wall, enzyme classification, phytohormones, protein modification and degradation, RNA biosynthesis, and solute transport were similarly relevant to *A. thaliana* and *S. viridis* but the number of DEGs and timing of changes were divergent. The subset of DEGs belonging to each bin category and plant species is provided in Supplementary Dataset 8. It is worth mentioning that the genome of *S. viridis* was relatively recently sequenced (Bennetzen et al., 2012), imposing limitations in its functional annotation. Nevertheless, we focused on contrasting responses to the aforementioned biological processes that somehow are independent of these factors.

Biosynthetic growth relies on C assimilation and metabolic conversion into macromolecules such as proteins, carbohydrates, and cell walls. Protein synthesis, essential for plant growth, is extremely costly in terms of ATP consumption for both amino acids synthesis and for elongating polypeptide chains (Piques et al., 2009). The main readout of TORC is the regulation of translation (Deprost et al., 2007; Sormani et al., 2007; Ahn et al., 2011; Ren et al., 2011, 2012; Schepetilnikov et al., 2011, 2013; Dobrenel et al., 2016b; Schepetilnikov and Ryabova, 2018). Not surprisingly, TOR repression massively down-regulated the transcription of a long list of ribosomal proteins and binding factors in *A. thaliana* plants, whose number of related DEGs was reduced in *S. viridis*. Not only RNA and protein biosynthesis are negatively affected by TOR inhibition, but also the transport of proteins to the endoplasmic reticulum is disturbed solely in *A. thaliana*. Large amounts of protein are invested in the synthesis of enzymes. DEGs related to enzymes are broadly affected in both species after treatment with AZD8055, reinforcing the role of TORC in controlling metabolism. However, those alterations were described linked to the main biological processes they belong to (e.g., cell walls). N is also required for the biosynthesis of nucleotides, chlorophyll, and several other metabolites essential for biosynthetic growth. Transcripts of transporters (e.g., amino acid permeases, LYS/HIS transporter, and peptide transporter 2, receptors (e.g., glutamate receptors), and enzymes (e.g., glutamate dehydrogenase 3 and tyrosine aminotransferase 3) involved in N metabolism were usually repressed in *S. viridis* and induced in *A. thaliana* after AZD8055 treatment. One exception was the high-affinity nitrate transporter *NRT2.1*, particularly effective when nitrate is limiting (Krapp, 2015). Genes involved in the metabolism of the phytohormone cytokinin (CK), closely associated with N availability and signaling (Sakakibara, 2021), are also altered in both species under TOR suppression. Together, these data support the higher N use efficiency of *S. viridis*, typical for C_4_, and suggests that N metabolism seems to be less dependent on TOR control in this species. There is a close relationship between N and C metabolism as substantial amounts of assimilated N are allocated into the enzymes of the photosynthetic apparatus that produces C skeletons for the basic building blocks of biomass accumulation (Nunes-Nesi et al., 2010).

Prolonged TORC inhibition (<24 h) leads to changes in many transcripts related to photosynthesis (Dong et al., 2015), but this does not seem to be the case for short-term TOR repression neither in *A. thaliana* nor *S. viridis* (Supplementary Dataset 8). Only nine genes encoding proteins from photosystem II and components of chloroplast NAD(P)H dehydrogenase complex are generally suppressed by AZD8055 in *A. thaliana*. In *S. viridis*, single genes are up- (transketolase) and down-regulated (unknown protein related to photosynthesis) after 6 h of treatment with AZD8055. However, transcripts related to carbohydrate transport (*SWEET*s) and metabolism (sucrose synthase, starch-degrading enzymes, *MIOX1*, and galactinol synthase) are affected by TORC inhibition but less tightly regulated in *S. viridis*. This transcriptional profile suggests a more intensively impaired capacity of adequate carbohydrate production and allocation to fuel growth in *A. thaliana*. This is supported by the increased starch levels (Supplementary Figure 5) that restrict C availability for growth, resulting in a more pronounced decrease in FW and DW in this species (Figure 3C, Supplementary Figure 3F).

Besides providing metabolic energy, C storage compounds or photoassimilates can be metabolized into UDP-sugars that are incorporated into the plant cell wall polysaccharides, which act as the main C sink to the cells. Despite the known substantial differences in grasses and eudicot cell walls, AZD8055 triggered not only species-specific changes but also common processes. One example is the repression of expansins (EXPAs), endotransglucosylases/hydrolases (XTH), extensins (EXT), and transcription factors (TFs) responsible for cell wall loosening and expansion in both species. In *S. viridis*, TORC inhibition seems to reduce the expression of genes associated with the cell wall polysaccharide synthesis (cellulose synthase and glycosyltransferases – GTs) or rearrangements (glycosyl hydrolases) together with laccase. Nonetheless, in *A. thaliana*, a higher number of genes related to pectin stiffening (e.g., pectin methylesterase and pectin lyases-like) and wall strengthening (e.g., EXT and proline-rich proteins) had lowered expression after AZD8055 treatment. Cell wall impairment caused by different actors probably converged to the reduced biomass (DW) and cell length observed in both species.

Whereas water influence is absent from DW measurements, this is not the case for FW (Huang et al., 2019). The impact of AZD8055 on *S. viridis* FW might be associated with the water dynamics. Whilst various aquaporin genes were down-regulated in *S. viridis*, such as plasma membrane intrinsic proteins (PIPs), only one tonoplast and one nodulin-26 like intrinsic proteins were affected in *A. thaliana* after TOR repression. Collective down-regulation of PIPs is described to reduce water loss (Afzal et al., 2016), although changes in aquaporin transcripts rely on several elements (e.g., isoform, tissue, species, and stimuli). In *A. thaliana*, TORC impairment seems to induce strategies of water stress responses that are regulated by other mechanisms, such as general TFs (*Myb41*, *Myb107*, *FAR5*, *KCS2*, and *CYP86B1*), root hair inducers (*Myb2*, *CPL3*, *ABF3*, and *RSL4*), and proteins related to suberin synthesis and transport, all up-regulated. The down-regulation of TOR switches not only water stress-tolerant genes (Bakshi et al., 2017b; Wang et al., 2018) but activates the genetic program of general stress responses such as NACs, MYBs, and WRKYs TFs that are mostly up-regulated in inhibited-TOR *A. thaliana. S. viridis* presented lesser stress response-DEGs, which showed mixed expression patterns.

Our transcriptomics data revealed a smaller magnitude of short-term TORC impairment on global gene expression in *S. viridis*. However, alterations in transcripts from C and N metabolism, particularly cell wall synthesis and protein translation, probably contribute to the reduced DW in this species.

## Discussion

Primary metabolism fuels plant growth and development through the synthesis or turnover of metabolites and storage compounds (Sulpice and McKeown, 2015). TORC plays an essential role in this process, acting as a nutrient/energy sensor and adjusting growth and metabolic activities accordingly (Wu et al., 2019; Liu and Sabatini, 2020). As autotrophic organisms, plants incorporate C through photosynthesis and its allocation is one of the key factors controlling biomass production. In addition to altering different aspects of photosynthesis such as non-photochemical quenching and electron transport (Upadhyaya and Rao, 2019) as well as CO_2_ assimilation rates (Salem et al., 2018), the TOR pathway was demonstrated to play a role in regulating the partitioning into photosynthetic end products (Jüppner et al., 2018; Salem et al., 2018; Pancha et al., 2019). In this study, we were interested in comparing how TORC disruption would affect a C_4_ grass model as a means to advance the understanding of metabolic control in a faster growth rate species. C_4_ faster growth rate is derived from the smaller tissue density that allows the production of more leaves and a higher investment in roots, increasing the efficiencies on water and nutrients uptake (Atkinson et al., 2016). Despite the differences in photosynthesis, *S. viridis* and *A. thaliana* present additional structural, morphological, and genetic specificities that have classified them into distinct clades named eudicots and monocots, respectively. Their roots differ in the number of quiescent and cortical cells, general root and hair patterns (Hochholdinger and Zimmermann, 2008), whereas their leaves diverge in the shape and direction of leaf veins and the temporal versus spatial growth leaf regulation (reviewed by Nelissen et al., 2016). Therefore, the effect of TORC repression on the metabolism and growth of *S. viridis* and *A. thaliana* has to be considered in a more comprehensive view as different requirements might be needed for TORC-mediated growth control in each species.

Protein sequence analysis pinpointed a high degree of conservation of TOR in the plant kingdom with few residue exchanges that might lead to specificities between monocots and eudicots (Supplementary Figure 1). In most land plants, reduced rapamycin sensitivity (Xu et al., 1998; Menand et al., 2002; Ren et al., 2012; Montané and Menand, 2013; Deng et al., 2017) was mainly attributed to point mutations at residues K-53, Q-54, and E-55 of the FKBP12 corresponding to the human sequence (Choi et al., 1996; Crespo et al., 2005; Mahfouz et al., 2006; Deprost et al., 2007; Sormani et al., 2007; Ren et al., 2012). *S. viridis* FKBP12 protein sequence presents residues L-53 and S-55 that differ from rapamycin-sensitive species (Supplementary Figure 2A). Alternatively, the monocots maize and rice both present corresponding residues Q-53 and S-55 but do respond differently to rapamycin (García Flores et al., 2001; Menand et al., 2002; Reyes de la Cruz et al., 2004; Agredano-Moreno et al., 2007). These results reinforce the limitations of using rapamycin as a TORC inhibitor among different plant species. Our data on yeast complementation suggest that the treatment with rapamycin has a slightly different effect between SvFKBP12 and AtFKB12. This could come from the different expression levels of these proteins in yeast or a slightly higher affinity of SvFKBP12 for rapamycin, which needs more detailed investigation. However, the formation of *S. viridis* ternary complex seems to be also unstable (Supplementary Figure 2). Nevertheless, the asTORis AZD8055 had a more pronounced effect on root growth reduction than rapamycin in this grass.

The temporal dynamics of TORC inhibition mediated by the chemical AZD8055, extensively employed to investigate the TOR pathway in plants (Montané and Menand, 2013; Dong et al., 2015; Ouibrahim et al., 2015; Dobrenel et al., 2016b; Prioretti et al., 2017; Schepetilnikov et al., 2017; Soprano et al., 2017; Inaba and Nagy, 2018; Mohammed et al., 2018; Riegler et al., 2018; Barrada et al., 2019; Forzani et al., 2019; Song et al., 2019; Van Leene et al., 2019; Méndez-Gómez et al., 2020; Zhu et al., 2020), was followed in seedlings of *S. viridis* and *A. thaliana* at specific and compatible developmental stages. Dose-dependent experiments, using the root GI_50_ as a parameter (Figure 2), revealed that growth arrest is a phenotypic consequence of TORC repression in both species. Not only root growth was restrained (i.e., root length and cell growth and proliferation) but FW and DW (Figure 3, Supplementary Figure 3). The smaller impact of TOR inhibition in *S. viridis* DW and length of EZ in roots suggests differential regulation of both biomass accumulation and cell elongation, essential for plant growth. The fact that C_4_ species display 19–88% daily DW increase compared to C_3_ depending on the plant size (Atkinson et al., 2016) and emerging evidence on distinct responses of TOR signaling to external stimuli (e.g., light) in C_4_ (Henry et al., 2020) might be relevant for this specificity. Factors such as complexity of root architecture, the kinetics of drug uptake and efflux, and metabolic mechanisms to detoxify part of the drug (Kreuz et al., 1996) could also further contribute to this reduced sensitivity in *S. viridis*, as it seems that the effect of the drug had a narrower window in this grass (Figure 3B, Supplementary Figure 3E). Nevertheless, distinct species requiring different asTORis doses can result in similar molecular and phenotypic responses of plant impaired development (reviewed by Montané and Menand, 2019).

In accordance with the phenotypic analysis, *S. viridis* presented milder alterations in primary metabolites and global gene expression under TORC repression by AZD8055 compared to *A. thaliana*. Numerous DEGs belonging to previously reported categories modulated by TORC were affected in both species (Figure 5B, Supplementary Dataset 7) (Deprost et al., 2007; Sormani et al., 2007; Ahn et al., 2011; Ren et al., 2011, 2012; Schepetilnikov et al., 2013; Dong et al., 2015; Dobrenel et al., 2016b; De Vleesschauwer et al., 2018; Forzani et al., 2019) and classical metabolic TORC signatures related to sugars, amino acids, and intermediates from the TCA cycle were recognized (Figure 4 and Supplementary Dataset 3) (Moreau et al., 2012; Caldana et al., 2013; Lee and Fiehn, 2013; Jüppner et al., 2018; Mubeen et al., 2018; Salem et al., 2018; Zhang et al., 2018).

The less pronounced inhibition of protein translation, which is the typical molecular phenotype of TOR repression, together with down-regulation of fewer genes related to N uptake and transport and minor magnitude of alterations in amino acids might indicate that TORC makes a less significant contribution to monitoring N status in *S. viridis*. On the other hand, N metabolism was more densely affected in *A. thaliana*. Arg, its precursors citrulline and Orn, and transcripts associated with Arg biosynthesis such as *BAC2* (Planchais et al., 2014) were markedly reduced during the day in this species under TORC inhibition (Figure 4 and Supplementary Dataset 3). These results are consistent with higher N demand. Arg functions as the major storage and transport form for organic N due to its increased N to C ratio (Schneidereit et al., 2006). Arg catabolism is also an artifact to mobilize N according to stress and nutritional limitations (Winter et al., 2015) and corroborates with the perturbations in C/N metabolism resulting from TOR inhibition (Ren et al., 2012; Caldana et al., 2013; Mubeen et al., 2019). Alternatively, the lower Arg and Orn in *A. thaliana* might be partially due to Proline (Pro) accumulation as they take part in Pro biosynthesis. Pro and its regulators *WRKY54* and *WRKY70* (Li et al., 2013) were also elevated in this species. Recently, high abundances of Pro and other amino acids were implicated in inhibited leaf respiration under TORC control at night-time (O’Leary et al., 2020). The *A. thaliana lst8-1* mutant also displays higher Pro content, which is amplified after transference from short to long days, and fails to produce raffinose and galactinol that replace Pro during this adaptation to longer light periods (Moreau et al., 2012). In our equinoctial conditions, galactinol and raffinose levels increased after treatment with AZD8055 in *A. thaliana*. These compounds are proposed as cellular membrane osmoprotectants and stabilizers, and ROS scavengers (Nishizawa-Yokoi et al., 2008a, b). ROS marker genes had their transcript levels augmented, which could be an indication of induced ROS production (Garcia et al., 2014), and correlate with the higher impairment of cell expansion (Dunand et al., 2007) in this eudicot. Our results also show a strong connection between TORC and CK, which overlaps in several aspects of plant growth (e.g., promotion of cell proliferation and differentiation, cell cycle regulation, and nutrient signaling). CK oxidases (*CKX1*, *CKX5*, and *CKX6*) that promote CK degradation were repressed in *S. viridis*, whereas CK hydroxylases (*CYP735A1* and *CYP735A2*) implicated in CK biosynthesis were inhibited in *A. thaliana* as well as *ABCG14*. It remains to be elucidated whether these observed changes in transcripts of CK metabolism would result in altered CK levels that modulate organ growth besides N acquisition and distribution (Rahayu et al., 2005; Werner and Schmülling, 2009; Ruffel et al., 2011; Landrein et al., 2018). Together, these alterations match higher N use efficiency (Ghannoum et al., 2011) in grasses that jointly with morphological features (e.g., crown root) contribute to the acquisition of adequate quantities of nutrients, faster growth rates, and higher biomass.

Regarding C metabolism, only a few genes related to photosynthesis were affected in both species in the short time frame of our experiment. It was recently shown that changes in light intensities broadly impact photosynthetic genes in *S. viridis* but transcripts of the TOR signaling pathway remained unaffected, suggesting a differential sugar feedback inhibition than C_3_ (Henry et al., 2020). In contrast, TOR and its downstream targets are post-translationally regulated. This might clarify why TORC disruption impacted only two photosynthetic transcripts in *S. viridis* (Supplementary Dataset 8). However, C metabolism and partitioning were altered at the transcriptional and metabolite level indicating that TORC may exert a differential control on C allocation in both species. Treatment with AZD8055 does not impact starch reserves in *S. viridis*, a classical readout of TORC inhibition *in A. thaliana* and algae (Caldana et al., 2013; Jüppner et al., 2018; Salem et al., 2018; Pancha et al., 2019). However, the levels of the organic acid fumarate decreased in *S. viridis* under TOR repression, which could limit the usage of this C skeleton to fuel growth. Fumarate together with malate represent an alternative C sink for photosynthate (Chia et al., 2000; Gibon et al., 2006; Fahnenstich et al., 2007; Pracharoenwattana et al., 2010) and have been addressed as storage C molecules (Zell et al., 2010). Their accumulation could be associated with reduced energy and ultimately growth in *A. thaliana* (Figure 4 and Supplementary Dataset 3). This species has incredibly high fumarate contents compared to other C_3_ (Araújo et al., 2011) and shows large diel fluctuations (Gibon et al., 2006; Sulpice et al., 2014). Fumarate is also involved in pH regulation during nitrate assimilation (Araújo et al., 2011). Transcripts encoding the enzyme *MIOX1* involved in myo-inositol metabolism crucial for nucleotide sugar biosynthesis of plant cell walls (Kanter et al., 2005) were up-regulated in *A. thaliana* after treatment with AZD8055. *MIOX1* is induced in C-starved *A. thaliana* seedlings and its expression together with other *MIOX* family members was proposed to allow scavenging of alternative C sources (Osuna et al., 2007). The augmented levels of glycerate in *A. thaliana*-treated plants (Figure 4 and Supplementary Dataset 4) might indicate higher photorespiration, in agreement with the up-regulation of some N transporters in this species. Photorespiration forces C_3_ to heavily invest in the production of RubisCO, making them more N demanding (Lara and Andreo, 2011). Increased Ser levels were observed in both species after AZD8055 treatment and might be associated with the transcriptional regulation of photorespiration (Timm et al., 2013). Interestingly, Ser biosynthesis through the phosphorylated pathway seems to be linked with S assimilation in C_4_ (Gerlich et al., 2018). S metabolism is also under TORC control (Dong et al., 2017) and two genes were commonly disturbed in both species but in opposite directions (i.e., positively and negatively affected in *A. thaliana* and *S. viridis*, respectively): sulfurtransferase 18 involved in cellular redox homeostasis (Henne et al., 2015) and phloem sulfate transporter 1.3 important for source to sink transport (Yoshimoto et al., 2003). The induction of *LSU1*, a marker for S deficiency, together with the repression of the sulfate transporter 1;1 responsible for sulfate uptake by roots (Yoshimoto et al., 2003) suggest S limitation after AZD8055 treatment in *A. thaliana*, which can imbalance S-containing amino acids like methionine (Figure 4) and impact growth. Among crops, members from the Brassicaceae family are more S-dependent than Poaceae (Aarabi et al., 2020), which might explain why S metabolism was more markedly affected in *A. thaliana.* TORC repression also disturbed the expression of genes involved in the metabolism of other nutrients, such as potassium, iron, zinc, and copper in both species but the relevance of those alterations to plant biomass accumulation needs further investigation.

Cell wall synthesis seems to be prioritized and regulated by C supply, representing the largest sink for photosynthetically fixed C and the driving force behind expansion- and biomass-based growth (DW) (Verbancic et al., 2018). As a master regulator of cell growth, it is not surprising that TOR repression leads to massive changes in cell wall genes (Moreau et al., 2012; Ren et al., 2012; Xiong and Sheen, 2012; Caldana et al., 2013; Dong et al., 2015). *A. thaliana* type I cell walls have similar levels of cellulose and xyloglucans embedded in pectin, whereas *S.viridis* type II presents lower abundances of structural proteins, xyloglucan, and pectin, but higher levels of phenylpropanoids and arabinoxylans (Vogel, 2008). The rapidly increase in xylose levels after AZD8055 treatment (Figure 4) indicate less incorporation of this sugar into xylan to compose the *S. viridis* hemicellulosic biomass, matching the down-regulation of GTs. Moreover, in *S. viridis*, reduced growth seems to be related to lowered primary and secondary cell wall polymers biosynthesis and modifications, as shown in our omics data. In *A. thaliana*, however, the down-regulation of genes related to pectin stiffening is in agreement with previous studies (Ren et al., 2012; Caldana et al., 2013; Xiong et al., 2013) suggesting that reduced TOR activity leads to altered pectin content (Leiber et al., 2010), impairing plant growth. In both species, the decreased DW may result from reduced cell wall synthesis controlled by specific mechanisms, from which we might relate the less incorporation of xylose in *S. viridis* and reduced pectin in *A. thaliana*.

Besides biomass incorporation, cell wall expansion is one of the main restrictions for plant cell growth. As some EXPAs and XTHs are root-specific, the downregulation of their expression probably contributed to the reduced root final cell size in both species (Supplementary Figures 3C,D). The differential number of water-related transcripts (such as PIPs) coupled to contrasting hydraulic conductance (Ghannoum et al., 2011; Taylor et al., 2012) might indicate a diverse impact on the turgor pressure needed for cell expansion. In *A. thaliana*, the up-regulation of stress-responsive TFs and suberin/lignin-related transcripts pinpoint that stress sensing was more heavily impacted and different strategies were adopted, such as avoiding water loss by suberization. Interestingly, ectopic overexpression of AtTOR in rice is described to potentially increase yield under water restriction (Bakshi et al., 2017a). The smaller root cell length in both species seems to be derived from impaired cell elongation provoked by the down-regulation of several enzymes, however insufficient turgor pressure suggested by the transcriptomics data may play an additive effect, leading to reduced FW. The mechanisms of water loss avoidance triggered by AZD8055 treatment may contribute to the changes observed in *S. viridis* FW and a smaller impact on root EZ length, as water influences cell elongation (Ivakov et al., 2017).

Cell division is the third axis that defines growth, besides biomass incorporation and cell expansion. The increased meristematic cell length coupled to the decrease in MZ cell number and length indicate repression of cell division in *S. viridis* roots. This is consistent with the up-regulation of NAC087 and NAC078, TFs related to cell death (Huysmans et al., 2018) and negative control of cell proliferation (Tang et al., 2016), respectively. In addition, *S. viridis* presented high levels of DHA that associated with the decreased transcript levels of the detoxifiers glutathione S transferases (GST) at 6 h might reflect an inadequate ROS protection (Miret and Müller, 2017). DHA can be recycled by the GST dehydroascorbate reductase (DHAR) to produce ascorbate (ASC), an important cell antioxidant involved in maintaining cellular redox homeostasis. Suppression of DHAR slowed down leaf expansion and DW in tobacco (Chen and Gallie, 2006) whilst DHA delayed cell cycle progression in maize and BY-2 tobacco cells (Kerk and Feldman, 1995; Potters et al., 2000). The accumulation of DHA coupled with a decrease in GSTs expression indicates that this compound might not be recycled to ASC, which could have negatively impacted *S. viridis* DW, root cell expansion and proliferation. Our data suggest that the lesser incorporation of xylose into *S. viridis* cell wall mediated by enzymes might be one of the reasons for decreased DW, together with impaired regeneration of ASC. The reduced expression of genes coding for enzymes that perform cell expansion (i.e., EXPAs) and rearrangements (i.e., GHs) are consistent with root cell length decrease. On the other hand, the suggested antioxidant impaired activity coupled to the regulation of specific transcription factors could have contributed to the *S. viridis* meristematic cell number reduction under TOR inhibition. However, target analyses are needed in order to tight the correlation of specific actors under TOR regulation with the observed phenotypic changes. Taken together, our results give further indication that TOR signaling coordinates the three aspects of plant growth; biomass accumulation, cell elongation and division, and strengthen the role of this pathway as a positive general cell wall regulator in plants, acting differentially in each cell wall type.

In conclusion, our data provide a glimpse of the major processes affected by TORC in a C_4_ model grass with fast growth rates. Improved nutrient use efficiency and C allocation and partitioning optimize *S. viridis* biosynthetic growth, which seems to be under less tight regulation of the TOR pathway. Besides photosynthetic differences, *S. viridis* and *A. thaliana* present several specificities that classify them into distinct lineages, which also contribute to the observed alterations mediated by TOR. Functional studies are required to further elucidate the mode-of-action of TOR in different plant species. Despite the similarities of TORC sequences, domains, and known phosphorylation sites, it remains to be investigated whether secondary targets could switch diverse genetic and metabolic networks. A better mechanistic analysis considering the sink and source tissues is needed to uncover to which extent these observations hold true.

## Data Availability Statement

RNA-seq datasets from this study can be found in the NCBI SRA database online repository under accession number BioProject ID PRJNA494848 (https://www.ncbi.nlm.nih.gov/bioproject/PRJNA494848). *S. viridis* FKBP12 complete cds sequence is available in the NCBI GenBank database under ID MN927224.1 (https://www.ncbi.nlm.nih.gov/nuccore/MN927224.1/).

## Author Contributions

VCHS, MCMM, and VM designed the experiments. MJC-R and VCHS analyzed protein sequences. VCHS and TJPS performed *in silico* ligand analyses. VCHS did yeast complementation tests. VCHS, CCMB, and VM carried out AZD8055 dose-response experiments. AA performed microscopy analysis. MCMM did growth experiments. CCMB and VM did RNA extraction and RNAseq library preparation and extracted metabolites. SG and DMR-P did RNAseq bioinformatics analysis. CC carried out metabolomics analyses and supervised all experiments and revised the manuscript. MCMM, MJC-R, VCHS, and CC interpreted data and wrote the manuscript. All authors contributed to the article and approved the submitted version.

## Conflict of Interest

The authors declare that the research was conducted in the absence of any commercial or financial relationships that could be construed as a potential conflict of interest.

## Footnotes

1 https://www.ncbi.nlm.nih.gov/

2 https://www.ebi.ac.uk/Tools/msa/clustalo/

3 https://www.genome.jp/tools/motif/

4 https://www.ebi.ac.uk/interpro/search/sequence/

5 https://www.r-project.org

6 https://plabipd.de/portal/mercator4

7 https://www.ncbi.nlm.nih.gov/sra

## Supplementary Material

Supplementary Figure 1 Predicted residues responsible for ATP binding and phosphorylation in TOR kinase sequences from photosynthetic species. Schematic TOR protein domain structure and multiple amino acid sequence alignment. Numbers indicate the amino acid position along the protein sequence. The alignment shows the kinase and FATC domains from *Arabidopsis thaliana* (Ath), *Solanum lycopersicum* (Sly), *Oryza sativa* (Osa), *S. viridis* (Svi), *Sorghum bicolor* (Sbi), *Zea mays* (Zma), and *Chlamydomonas reinhardtii* (Cre). Conserved residues, essential for ATP binding, are marked in red. Residues implied in mLST8 interaction are shown in blue, whereas residues implied in kinase activity are displayed in green and yellow. The TOR activation phospho-site corresponding to S-2424 in Arabidopsis is shown in magenta.

Supplementary Figure 2 Analysis of TOR-RAPAMYCIN-FKBP12 ternary complex in *S. viridis*. **(A)** Amino acid sequences of FKBP12 from *S. viridis* (Svi) compared to other representative organisms: *Sorghum bicolor* (Sbi), *Zea mays* (Zma), *Arabidopsis thaliana* (Ath), *Solanum lycopersicum* (Sly), *Oryza sativa* (Osa), *Chlamydomonas reinhardtii* (Cre), *Saccharomyces cerevisiae* (Sce), *Caenorhabditis elegans* (Cel), *Drosophila melanogaster* (Dme), and *Homo sapiens* (Hsa). Identical residues are shaded in gray, and identity percentages are indicated aside. Red asterisks indicate residues involved in rotamase activity in non-photosynthetic organisms (DeCenzo et al., 1996). Blue asterisks indicate two cysteine residues at fixed positions, characteristic of plant sequences. Red boxes indicate residues from human FKBP12 that interact with rapamycin (Choi et al., 1996). The green bar indicates the rapamycin binding regions of HsaFKBP12. **(B)** Yeast complementation assay performed in solid media supplemented with rapamycin concentrations ranging from 0–1 μM. Wild-type BY4741 cells were transformed with the empty vector (WT + P). The *fpr1* mutant strain lacking the FKBP12 protein was transformed with the empty vector (*fpr1*Δ + P) or with recombinant plasmids expressing FKBP12 from *S. cerevisiae* (ScFKB12), *A. thaliana* (AtFKBP12), or *S. viridis* (SvFKBP12). Cultures were normalized at OD_600_ = 0.6, subjected to 10-fold serial dilutions, and spotted onto selective SD-Ura plates containing different concentrations of rapamycin or the diluent 0.1% (90% ethanol, 10% Tween20^(R)^). Plates were incubated at 30°C for 2 days. **(C)** The logarithmic growth phase, assessed up to 10 h, in SD-Ura liquid media supplemented with 1 μM rapamycin was adjusted by linear regression to obtain the slope value of each strain to measure their doubling times. The slope values were obtained by plotting log_10_OD_600_ in the function of time and adjustment of the linear regression equation *y* = ax + b, where *a* = slope, *b* = intercept, when *X* is 0. **(D)** Doubling times peak in yeast strains grown under liquid cultures supplemented with 1 μM were determined by the equation Td = log_2_/a, where Td is the time of duplication, and “a” is the slope determined by linear regression equation of log phase. At least three colonies per strain were analyzed for each treatment. The graphs and statistics were obtained with OriginPro (version 8.5). Significant differences comparing every strain using ANOVA are indicated by letters (*P* < 0.05). **(E)** Effect of rapamycin and AZD8055 on root growth of *S. viridis* along 7 days of treatment. Seedlings were grown under a 12 h photoperiod until reaching stage 11 (BBCH scale), when they were transferred to plates containing DMSO 0.05% (control), AZD8055 (10 μM) or rapamycin (10 μM).

Supplementary Figure 3 Treatment with AZD8055 severely impacts root and overall growth of *S. viridis* and *A. thaliana* seedlings. Seedlings of *S. viridis* and *A. thaliana* were grown under 12 h photoperiod until specific and compatible developmental stages before the transference to plates containing AZD8055. The effect of different concentrations of AZD8055 (1, 2 and10 μM) or DMSO 0.05% (control) on root growth of *S. viridis* **(A)** and *A. thaliana* **(B)** was expressed in cm along days of treatment. For the root zone and cell length measurements, 10 and 2 μM AZD8055 was used for *S. viridis* and *A. thaliana*, respectively. **(C)** Length of meristematic cells. **(D)** Length of mature cells. Fresh weight of *S. viridis* **(E)** and *A. thaliana* **(F)**. **(G)** Estimative of the number of cells at the meristematic zone of *S. viridis* and *A. thaliana*.

Supplementary Figure 4 Principal component analysis (PCA) of primary metabolic profiles of *S. viridis* and *A. thaliana* upon TORC-inhibition. Seedlings were grown hydroponically under 12 h photoperiod until specific and compatible developmental stages and exposed to treatment with DMSO 0.05% (control), 10 or 2 μM AZD8055 (*S. viridis* and *A. thaliana*, respectively). Metabolite profiling was carried out using GC-TOF-MS. **(A)** *A. thaliana*. **(B**) *S. viridis*. Blue and red colors represent DMSO and AZD8055 treatments, respectively, whereas gray indicates the beginning of the treatments (0 h, ZT 23,5).

Supplementary Figure 5 Starch content in *S. viridis* and *A. thaliana* seedlings under DMSO 0.05% (control), 10 or 2 μM AZD8055 treatment, respectively.

Supplementary Table 1 Protein IDs used for the analyses of TOR sequences.

Supplementary Table 2 Protein IDs of FKBP12 used for sequence alignment.

Supplementary Table 3 Primers for cloning full-length cDNA sequences of FKBP12 from *Saccharomyces cerevisiae* (ScFKBP12), *A. thaliana* (AtFKBP12), and *S. viridis* (SvFKBP12).

Supplementary Dataset 1 GC-TOF-MS measurements of polar metabolites from the MTBE-extraction in *S. viridis* and *A. thaliana* after AZD8055 treatment.

Supplementary Dataset 2 Principal component (PC) analysis of metabolites and their loadings contribution.

Supplementary Dataset 3 Overview of the metabolites differentially regulated in control and AZD8055 treatments in *S. viridis* and *A. thaliana*.

Supplementary Dataset 4 Summary of results obtained by RNAseq sequencing during short-term TORC repression in *S. viridis* and *A. thaliana*.

Supplementary Dataset 5 List of DEGs (log_2_FC ≥ 1 and FDR < 0.05) in distinct pairwise combinations in *S. viridis* during short-term TORC repression.

Supplementary Dataset 6 List of DEGs (log_2_FC ≥ 1 and FDR < 0.05) in distinct pairwise combinations in *A. thaliana* during short-term TORC repression.

Supplementary Dataset 7 Summary of DEGs in *S. viridis* and *A. thaliana* under TORC repression.

Supplementary Dataset 8 List of genes for each Mapman bin comparing AZD8055 vs DMSO treatments in *S. viridis* and *A. thaliana*.

Supplementary Dataset 9 Comparison of *A. thaliana* DEGs (| log_2_FC| > 1) with similar expression pattern among our data and previous studies (Ren et al., 2012; Xiong et al., 2013; Dong et al., 2015).
