# Supplementary material for: Shedding Light on the Dynamic Role of the “Target of Rapamycin” Kinase in the Fast-Growing C4 Species Setaria viridis, a Suitable Model for Biomass Crops
Source: Front Plant Sci. 2021 Apr 13;12:637508. doi: 10.3389/fpls.2021.637508 (PMC8078139; doi:10.3389/fpls.2021.637508)
Supplement: Supplementary Table 1 — Protein IDs used for the analyses of TOR sequences. [file Table_1.docx]

Supplementary Table S1. Protein IDs used for the analyses of TOR sequences.

| Organism/Abbreviation | TOR |
| --- | --- |
| *Arabidopsis thaliana* (Ath) | NP_175425.2 |
| *Solanum lycopersicum* (Sly) | XP_004230675.1﻿ |
| *Oryza sativa* subsp. *japonica* (Osa) | XP_015639567.1 |
| *Setaria viridis* (Svi) | Sevir.3G281300.1 |
| *Sorghum bicolor* (Sbi) | XP_021303451.1 |
| *Zea mays* (Zma) | NP_001105293.1 |
| *Chlamydomonas reinhardtii* (Cre) | ABB13529.1 |
