# Supplementary material for: Shedding Light on the Dynamic Role of the “Target of Rapamycin” Kinase in the Fast-Growing C4 Species Setaria viridis, a Suitable Model for Biomass Crops
Source: Front Plant Sci. 2021 Apr 13;12:637508. doi: 10.3389/fpls.2021.637508 (PMC8078139; doi:10.3389/fpls.2021.637508)
Supplement: Supplementary Table 2 — Protein IDs of FKBP12 used for sequence alignment. [file Table_2.docx]

Supplementary Table S2. Protein IDs of FKBP12 used for sequence alignment.

| Classification | Organism/Abreviation | FKBP12 | Rapamycin sensitivity (References) |
| --- | --- | --- | --- |
| C_3_ | *Arabidopsis thaliana* (Ath) | NP_201240.1 | Low (Ren *et al*., 2012; Xiong and Sheen, 2012; Deng *et al.,* 2016) |
| C_3_ | *Solanum lycopersicum* (Sly) | NP_001233825.1 | High (Xiong *et al*., 2016) |
| C_3_ crop | *Oryza sativa* subsp. *japonica* (Osa) | XP_015625368.1 | Low (Menand *et al*., 2002) |
| C_4_ grass | *Setaria viridis* (Svi) | MN927224.1 | Low (this work) |
| C_4_ crop | *Sorghum bicolor* (Sbi) | XP_002454586.1 | - |
| C_4_ crop | *Zea mays* (Zma) | NP_001105537.1 | High (Agredano-Moreno *et al.*, 2007) |
| algae | *Chlamydomonas reinhardtii* (Cre) | XP_001693615.1 | High (Crespo *et al.*, 2005) |
| yeast | *Saccharomyces cerevisiae* (Sce) | NP_014264.1 | High (Saxton and Sabatini, 2017) |
| nematode | *Caenorhabditis elegans* (Cel) | NP_001021722.1 | High (Saxton and Sabatini, 2017) |
| fruit fly | *Drosophila melanogaster* (Dme) | CAA88904.1 | High (Saxton and Sabatini, 2017) |
| mammal | *Homo sapiens* (Hsa) | NP_000792.1 | High (Saxton and Sabatini, 2017) |

- indicates that information is lacking.
