# Supplementary material for: Shedding Light on the Dynamic Role of the “Target of Rapamycin” Kinase in the Fast-Growing C4 Species Setaria viridis, a Suitable Model for Biomass Crops
Source: Front Plant Sci. 2021 Apr 13;12:637508. doi: 10.3389/fpls.2021.637508 (PMC8078139; doi:10.3389/fpls.2021.637508)
Supplement: Supplementary Table 3 — Primers for cloning full-length cDNA sequences of FKBP12 from Saccharomyces cerevisiae (ScFKBP12), A. thaliana (AtFKBP12), and S. viridis (SvFKBP12). [file Table_3.docx]

Supplementary Table S3. Primers for cloning full-length cDNA sequences of FKBP12 from *Saccharomyces cerevisiae* (ScFKBP12), *Arabidopsis thaliana* (AtFKBP12), and *Setaria viridis* (SvFKBP12).

| Primer | Sequence 5´-->3´ |
| --- | --- |
| Sce_FKBP12_Fwd | ATGTCTGAAGTAATTGAAG |
| Sce_FKBP12_Rev | TTAGTTGACCTTCAACAATTC |
| Ath_FKBP12_Fwd | ATGGGTGTGGAGAAGCAAG |
| Ath_FKBP12_Rev | TTACTGCACGCTCAG |
| His_SceFKBP12_pWS28_Fwd | ATTATCTACTTTTTACAACAAATCTAGAATTCCTGCAGCatgCATCATCACCATCACCACatgtctgaagtaattgaag |
| SceFKBP12_pWS28_Rev | TCCACCGCGGTGGCGGCCGCTCTAGAACTAGTGGATCCCttagttgaccttcaacaa |
| His_AthFKBP12_pWS28_Fwd | ATTATCTACTTTTTACAACAAATCTAGAATTCCTGCAGCatgCATCATCACCATCACCACatgggtgtggagaagcaag |
| AthFKBP12_pWS28_Rev | TCCACCGCGGTGGCGGCCGCTCTAGAACTAGTGGATCCCttactgcacgctcag |
