# Supplementary material for: Shedding Light on the Dynamic Role of the “Target of Rapamycin” Kinase in the Fast-Growing C4 Species Setaria viridis, a Suitable Model for Biomass Crops
Source: Front Plant Sci. 2021 Apr 13;12:637508. doi: 10.3389/fpls.2021.637508 (PMC8078139; doi:10.3389/fpls.2021.637508)
Supplement: Supplementary file 10 [file Data_Sheet_2.docx]

**Supplementary Figures**

**Supplementary Figure S1. Predicted residues responsible for ATP binding and phosphorylation in TOR kinase sequences from photosynthetic species.**

Schematic TOR protein domain structure and multiple amino acid sequence alignment. Numbers indicate the amino acid position along the protein sequence. The alignment shows the kinase and FATC domains from *Arabidopsis thaliana* (Ath)*, Solanum lycopersicum* (Sly), *Oryza sativa* (Osa), *S. viridis* (Svi)*, Sorghum bicolor* (Sbi), *Zea mays* (Zma), and *Chlamydomonas reinhardtii* (Cre). Conserved residues, essential for ATP binding, are marked in red. Residues implied in mLST8 interaction are shown in blue, whereas residues implied in kinase activity are displayed in green and yellow. The TOR activation phospho-site corresponding to S-2424 in Arabidopsis is shown in magenta.

**Supplementary** **Figure S2. Analysis of TOR-RAPAMYCIN-FKBP12 ternary complex in *S. viridis.***

**A.** Amino acid sequences of FKBP12 from *S. viridis* (Svi) compared to other representative organisms: *Sorghum bicolor* (Sbi), *Zea mays* (Zma), *Arabidopsis thaliana* (Ath), *Solanum lycopersicum* (Sly), *Oryza sativa* (Osa), *Chlamydomonas reinhardtii* (Cre), *Saccharomyces cerevisiae* (Sce), *Caenorhabditis elegans* (Cel), *Drosophila melanogaster* (Dme), and *Homo sapiens* (Hsa). Identical residues are shaded in gray, and identity percentages are indicated aside. Red asterisks indicate residues involved in rotamase activity in non-photosynthetic organisms (DeCenzo et al., 1996). Blue asterisks indicate two cysteine residues at fixed positions, characteristic of plant sequences. Red boxes indicate residues from human FKBP12 that interact with rapamycin (Choi et al., 1996). The green bar indicates the rapamycin binding regions of HsaFKBP12. **B**. Yeast complementation assay performed in solid media supplemented with rapamycin concentrations ranging from 0-1μM. Wild-type BY4741 cells were transformed with the empty vector (WT+P). The *fpr1* mutant strain lacking the FKBP12 protein was transformed with the empty vector (*fpr1Δ*+P) or with recombinant plasmids expressing FKBP12 from *S. cerevisiae* (ScFKB12), *A. thaliana* (AtFKBP12), or *S. viridis* (SvFKBP12). Cultures were normalized at OD_600_ = 0.6, subjected to 10-fold serial dilutions, and spotted onto selective SD-Ura plates containing different concentrations of rapamycin or the diluent 0.1% (90% ethanol, 10% Tween20^®^). Plates were incubated at 30 ^o^C for 2 d. **C.** The logarithmic growth phase, assessed up to 10 h, in SD-Ura liquid media supplemented with 1 µM rapamycin was adjusted by linear regression to obtain the slope value of each strain to measure their doubling times. The slope values were obtained by plotting log_10_OD_600_ in the function of time and adjustment of the linear regression equation y = ax + b, where a = slope, b = intercept, when X is 0. **D.** Doubling times peak in yeast strains grown under liquid cultures supplemented with 1 µM were determined by the equation Td = log_2_/a, where Td is the time of duplication, and “a” is the slope determined by linear regression equation of log phase. At least three colonies per strain were analysed for each treatment. The graphs and statistics were obtained with OriginPro (version 8.5). Significant differences comparing every strain using ANOVA are indicated by letters (*P* < 0.05). **E.** Effect of rapamycin and AZD8055 on root growth of *S. viridis* along 7 d of treatment. Seedlings were grown under a 12 h photoperiod until reaching stage 11 (BBCH scale), when they were transferred to plates containing DMSO 0.05 % (control), AZD8055 (10 µM) or rapamycin (10 µM).

**Supplementary Figure S3.** **Treatment with AZD8055 severely impacts root and overall growth of *S. viridis* and *A. thaliana* seedlings.**

Seedlings of *S. viridis* and *A. thaliana* were grown under 12 h photoperiod until specific and compatible developmental stages before the transference to plates containing AZD8055. The effect of different concentrations of AZD8055 (1, 2, and 10 µM) or DMSO 0.05% (control) on root growth of *S. viridis* **A.** and *A. thaliana* **B**. was expressed in cm along days of treatment. For the root zone and cell length measurements, 10 or 2 µM AZD8055 was used for *S. viridis* and *A. thaliana*, respectively. **C**. Length of meristematic cells. **D.** Length of mature cells. Fresh weight of *S. viridis* **E.** and *A. thaliana* **F**. **G.** Estimative of the number of cells at the meristematic zone of *S. viridis* and *A. thaliana*.

**Supplementary Figure S4. Principal component analysis (PCA) of primary metabolic profiles of *S. viridis* and *A. thaliana* upon TORC-inhibition.**

Seedlings were grown hydroponically under 12 h photoperiod until specific and compatible developmental stages and exposed to treatment with DMSO 0.05 % (control), 10 or 2 µM AZD8055 for *S. viridis* and *A. thaliana*, respectively. Metabolite profiling was carried out using GC-TOF-MS. **A**. *A. thaliana*. **B**. *S. viridis*. Blue and red colors represent DMSO and AZD8055 treatments, respectively, whereas grey indicates the beginning of the treatments (0 h, ZT 23,5).

**Supplementary Figure S5.** **Starch content in *S. viridis* and *A. thaliana* seedlings under DMSO 0.05 % (control), 10 or 2 µM AZD8055 treatment, respectively.**
